# Immature Teratoma of Nasal Septum: A Case Report 

**Published:** 2018-11

**Authors:** Subhro Ganguly, Surendra Gawarle, Prashant Keche

**Affiliations:** 1 *Department of Otorhinolaryngology, Topiwala National Medical College and BYL Nair Hospital, Mumbai, Maharashtra, India. *; 2 *Department of Otorhinolaryngology, Shri Vasantrao Naik Government Medical College, Yavatmal, Maharashtra, India. *; 3 *Department of Otorhinolaryngology, Aurangabad Government Medical College, Aurangabad, Maharashtra, India. *

**Keywords:** Chemotherapy, Endoscopic management, High-grade tumor, Immature teratoma, Nasal septum

## Abstract

**Introduction:**

Teratomas are neoplastic tumors derived from totipotent germ cells containing a wide assortment of tissues originating from all three germ cell layers. Teratomas can be mature or immature depending on the presence of immature tissues; typically neuroepithelial tissue. Immature teratomas can be oncologically benign or malignant, and can be divided into three grades with increasingly aggressive biological behavior. The most common site for this tumor is the sacrococcygeal region. The nasal septum is an exceptionally rare site for immature teratomas, with very few cases reported.

**Case Report:**

We discuss a 14-year-old male patient with a left nasal mass which, on histopathological examination, turned out to be a Grade-3 immature teratoma. Imaging revealed the mass to be confined in the left nasal cavity with erosion of the anterior skull base. During endoscopic excision, the tumor was seen extending intracranially but remaining extradurally. Complete resection was achieved, albeit with mild cerebrospinal fluid (CSF) leakage, which was closed successfully. The patient was subjected to adjuvant chemotherapy. A regular follow-up of 2 years showed no recurrence.

**Conclusion:**

The purpose of this report is to document the first case of a high-grade immature teratoma arising from the nasal septum with intracranial extension, as well as the efficacy of combined endoscopic resection and adjuvant chemotherapy for this pathology.

## Introduction

Teratomas are congenital or developmental tumors which are derived from multi-potent cells and differentiate into diverse types of tissue, representative of all three germ layers (1). Teratomas are rare tumors, accounting for only 3% of all childhood tumors, with sacrococcygeal teratoma being the most common (57%) followed by gonadal teratoma (29%). Teratomas of the head and neck region are exceedingly rare and represent only 2% of all teratomas (2,3). The most common site includes the neck, oropharynx, nasopharynx, orbit, and paranasal sinuses. In the sino-nasal tract, maxillary sinus and nasal cavity are mostly affected (4). Histologically teratomas can be mature, immature, or monodermal. Mature teratomas are typically benign, whereas immature teratomas can be oncologically benign or may contain malignant components and can behave aggressively. Thürlbeck and Scully first described the immature teratoma in 1960. The World Health Organization defines an immature teratoma as a teratoma consisting of a variable amount of immature embryonal tissues, generally neuroectodermal tissue (5,6).

Here, we discuss a case of immature teratoma (Grade 3) arising from the nasal septum in a 14-year-old boy, with emphasis on management and a brief review of literature.

## Case Report

A 14-year-old boy visited the ear, nose, and throat (ENT) outpatient department of our center, with a history of left-sided nasal obstruction gradually progressive over a period of 1 year, with recurrent history of associated rhinorrhea. There was no history of epistaxis or any other ENT complaints, and the patient’s past medical history and family history were insignificant.

**Fig 1:**
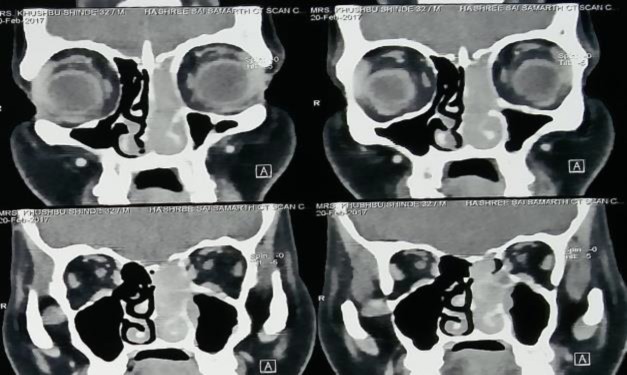
Contrast enhanced CT scan, coronal view showing contrast enhanced soft tissue mass in left nasal cavity with cribriform plate erosion

On anterior rhinoscopy, a single polypoidal mass was seen filling the left nasal cavity extending up to the vestibule. The mass was firm in consistency, insensitive to touch and pain, and did not bleed upon touch. General and systemic examinations and an examination of the rest of the ENT were all within normal limits. A contrast-enhanced computed tomography (CT) scan showed a homogenous soft tissue lesion measuring 44×68×12 mm in the left nasal cavity, extending posteriorly into the nasopharynx (Fig.1). 

**Fig 2:**
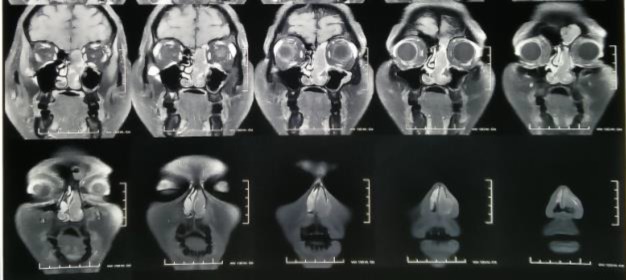
Post-contrast fat saturated T1w image coronal view showing soft tissue mass in left nasal cavity with cribriform plate erosion

The mass extended superiorly into the left ethmoid air cells up to the cribriform plate with its thinning and breach. The rest of the sinuses was clear. On contrast study, there was heterogeneous enhancement. Based on the CT findings of the anterior skull base breach, gadolinium-enhanced magnetic resonance imaging (MRI) was performed to rule out intracranial extension, and revealed small sub-centimeter focus posteriorly without any significant intracranial extension (Fig.2). 

Rigid endoscopy was carried out and the mass was seen attached to the anterior part of the nasal septum. A punch biopsy was taken, which presented as an inflammatory polyp.

**Fig 3:**
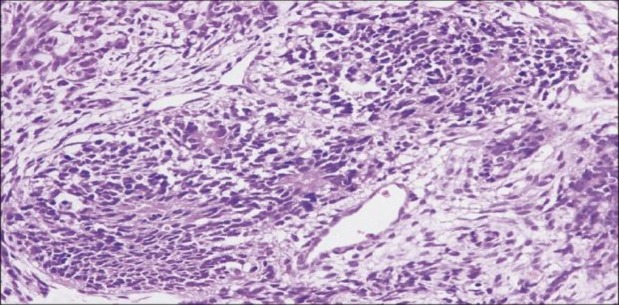
Histopathology showing primitive neuroepithelial cells forming rosettes (H and E stain, ×20)

**Fig 4:**
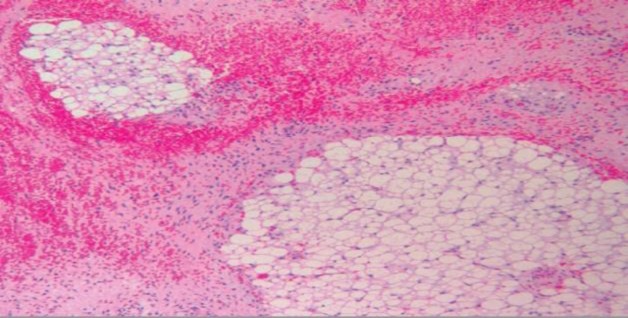
Histopathology showing clusters of mature adipose tissue with areas of hemorrhage and intervening stroma (H and E stain, ×10)

**Fig 5:**
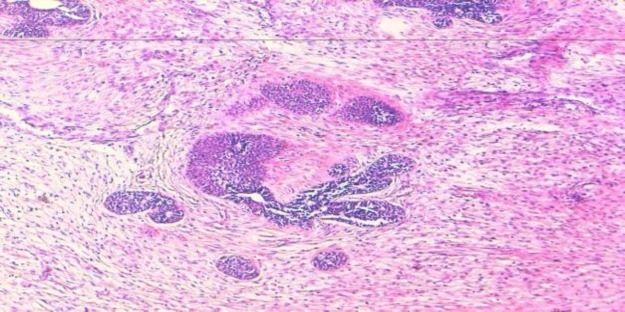
Histopathology showing neuroglial tissue with few glandular component (H and E stain, ×10)

**Fig6:**
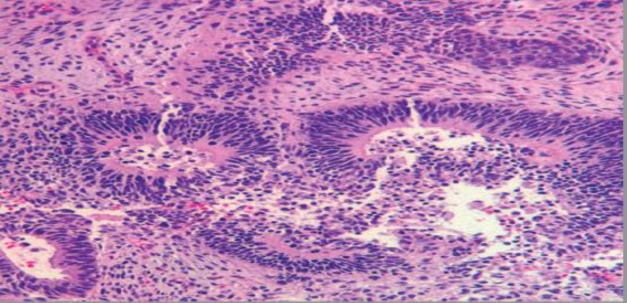
Histopathology showing Immature neuroectodermal elements (H and E stain, ×10

The patient was referred for standard endoscopic sinus surgery under general anesthetic after providing informed consent. The mass was removed endoscopically, and was seen to be extending intracranially but extradurally. Complete resection of the tumor was achieved and while managing the tumor in the region of the cribriform plate, there was an obvious CSF leak. The site of leakage was identified and closed using an underlay technique with septal cartilage, fascia lata, and tissue glue. The intra-operative blood loss was minimal.The post-operative recovery period was uneventful. Histopathology of the excised specimen showed the tumor mass lined by pseudostratified columnar epithelium with squamous metaplasia. The subepithelial tissue was composed of loose myxoid areas with mature adipose tissues and foci of cartilage. Foci of neuroepithelial cells occupying more than four low-power fields were seen. Grade 3 immature teratoma was given as final diagnosis (Fig. 3-6). 

Adjuvant chemotherapy was started based on the histological grading, with four cycles of bleomycin, etoposide, and cisplatin. A regular follow-up for a period of 2 years showed no signs of recurrence.

## Discussion

Teratomas are tumors of totipotent cells, consisting of mature and immature tissues originating from all three germ cell layers. The predominant component is ectodermal, which usually consists of neural tissue, teeth, skin, and hair. The mesodermal tissue is represented by fat, bone, and cartilage, whereas the endodermal component includes respiratory or intestinal epithelium. Primitive neuroepithelium with rosettes, pseudorosettes, or neurofibrillary matrix are observed in some tumors (1). Immature teratomas contain poorly-differentiated cells and are usually solid-nodular or solid-cystic. While cystic teratomas are chiefly benign, the solid teratomas are predominantly malignant (5,6).

The histogenesis of the head and neck teratoma is controversial, and the most accepted theory is displaced totipotent germ cells during embryogenesis and subsequent differentiation into teratomas (7). Head and neck teratomas seldom occur outside the neonatal period and are usually oncologically benign, while adult teratomas are usually malignant.

Unlike teratomas of other regions, which are more common in females, head and neck teratomas do not have a gender predilection (8). We encountered the pathology in a 14-year-old male patient as evident by other studies (5,7). Patients with sino-nasal teratomas usually present with vague symptoms such as nasal obstruction or rhinorrhea. One study reported a patient presenting with diplopia and proptosis (5). A CT scan is performed to investigate the extent of the tumor and its relationship to the adjacent structures. Although it does not differentiate between benign and malignant teratomas, a CT scan helps to identify solid and cystic components in a tumor. In 16% of cases, calcification is seen due to the presence of highly mineralized tissue, such as teeth or bone (5). MRI is also helpful in cases where the tumor has intracranial or intraorbital extension. In our case, the CT scan revealed erosion of the cribriform plate, and hence MRI was carried out to rule out intracranial extension. 

Another case study reported erosion of lamina papyracea with intraorbital extension (6).

Immature teratomas are histologically graded into three subtypes, with worsening prognosis, based on the amount of immature neuroectodermal tissues. If the foci of the neuroepithelial cells occupy no more than one low-power field per slide then it is characterized as Grade 1, while Grade 2 is established if the foci occupy no more than three low-power fields per slide. When the foci of the neuroepithelial cells occupy at least four low-power fields per slide then this is described as Grade 3 immature teratoma (9). 

It is necessary to at least identify tissues originating from two out of three germ layers; the ectoderm, mesoderm, and endoderm (1).

Complete surgical excision is the treatment of choice for sino-nasal teratomas irrespective of their histological nature, and the approach depends on the size of the tumor and its extent. Sreetharan et al. employed a lateral rhinotomy approach in their first documented case of teratoma of the nasal septum (7). In our case, the tumor was confined to the left nasal cavity with minimal breach of the cribriform plate, and hence an endoscopic approach was employed. 

This is a far superior modality compared with an open approach, as the associated angled telescopes and magnification help ensure complete removal of the tumor from every corner. Furthermore, this approach is associated with less intra-operative bleeding, shorter hospital stays, fewer surgical morbidities, and excellent cosmetic acceptability. Use of powered instruments such as a micro-debrider and electric scalpel has also been reported (5,10). Tumors with significant intracranial extension might require a combined endoscopic and neurosurgical approach (2). In 2011, Schuster reported the successful endoscopic resection of two intracranial dermoids with large anterior frontal lobe extensions using a 70-degree endoscope and a pedicled nasoseptal flap to close the skull base defect (11). Despite achieving complete resection, sino-nasal teratomas have high mortality rates (12). For this reason, a close follow-up is required; both radiological and endoscopic. Recurrence can be also detected by raised alpha-fetoprotein (AFP) levels since all types of teratomas are known to secrete AFP (7). Teratomas are highly chemo-sensitive and this modality is reserved for advanced tumors and relapse cases. Grade 2 and 3 teratomas should be treated with adjuvant chemotherapy, with four to six cycles of a bleomycin, etoposide, and cisplatin regimen in addition to surgery in order to achieve a long disease-free survival (13,14). We followed this protocol with regular endoscopic, radiological, and serological follow-up for 2 years with no signs of recurrence.

We conclude that sino-nasal teratoma, although an exceptionally rare pathology, can originate from the nasal septum, and this tumor should be included in the differential diagnosis of unilateral benign nasal tumors. The first documented case of nasal septal teratoma was reported in 2004 (7), and very few have been reported subsequently (5,12). All reported cases were nevertheless mature teratoma.

After a MEDLINE® search of the available literature, we failed to find any case report describing such advanced-stage immature teratoma of the nasal septum causing bony erosion of the skull base with intracranial extension. We further conclude that endoscopic resection with adjuvant chemotherapy is a safe option for these tumors, despite having a high recurrence rate and significant mortality.
